# Initial Response After Switching to Aflibercept 8 mg for Neovascular Age-Related Macular Degeneration

**DOI:** 10.3390/jcm14144824

**Published:** 2025-07-08

**Authors:** Chikako Hara, Satoko Fujimoto, Yoko Fukushima, Kaori Sayanagi, Kentaro Nishida, Kazuichi Maruyama, Shigeru Sato, Takatoshi Maeno, Kohji Nishida

**Affiliations:** 1 Department of Ophthalmology, Graduate School of Medicine, University of Osaka, Osaka 565-0871, Japan; satoko.fujimoto@ophthal.med.osaka-u.ac.jp (S.F.); youko.fukushima@ophthal.med.osaka-u.ac.jp (Y.F.); kaori.sayanagi@ophthal.med.osaka-u.ac.jp (K.S.); nishiken@ophthal.med.osaka-u.ac.jp (K.N.); kazuichi.maruyama@ophthal.med.osaka-u.ac.jp (K.M.); s.sato@ophthal.med.osaka-u.ac.jp (S.S.); takatoshi.maeno@ophthal.med.osaka-u.ac.jp (T.M.); knishida@ophthal.med.osaka-u.ac.jp (K.N.); 2 Department of Vision Informatics Integrated Frontier Research for Medical Science Division, Institute for Open and Transdisciplinary Research Initiatives, University of Osaka, Suita 565-0871, Japan

**Keywords:** aflibercept 8 mg, neovascular age-related macular degeneration, aflibercept 2 mg, faricimab, brolucizumab, exudative change

## Abstract

**Objectives**: This study aimed to assess the initial anatomical and functional outcomes of switching to aflibercept 8 mg in patients with neovascular age-related macular degeneration (nAMD) previously treated with anti-vascular endothelial growth factor (VEGF) therapy. **Methods**: Patients with nAMD previously treated with anti-VEGF drugs were switched to aflibercept 8 mg. Patients with any exudative changes (subretinal fluid [SRF], intraretinal fluid [IRF] or serous pigment epithelial detachment [sPED]) at the time of the first aflibercept 8 mg injection and whose dosing interval before and after switching did not differ by more than ±7 days were included. Best-corrected visual acuity (BCVA), central foveal thickness (CFT), and the presence of SRF, IRF, and sPED were evaluated before and after switching to aflibercept 8 mg. **Results***:* A total of 102 eyes from 98 patients were included in the analysis. The drugs used prior to switching were faricimab in five eyes, brolucizumab in six eyes, and aflibercept 2 mg in 91 eyes, with a mean interval of 63.7 ± 20.0 days from the last pre-switch injection and 64.9 ± 20.1 days to the first post-switch visit. The CFT was significantly reduced from 272 ± 85 µm to 246 ± 79 µm (*p* < 0.0001). The BCVA remained unchanged at 0.27 ± 0.35 logMAR. During switching, SRF, IRF, and sPED were observed in 70, 24, and 38 eyes, respectively. At the post-switch visit, complete resolution of exudative changes was observed in 44% of eyes with SRF, 55% with IRF, and 29% with sPED. No ocular or systemic adverse effects were observed. **Conclusions**: As an initial response to switching to aflibercept 8 mg in a real-world setting, SRF and IRF completely resolved in approximately half of the patients, and sPED resolved in about 30% of cases.

## 1. Introduction

Worldwide, neovascular age-related macular degeneration (nAMD) is a leading cause of central blindness in older adults [1,2,3]. Intravitreal vascular endothelial growth factor (VEGF) therapy is currently the first-line treatment for nAMD [4]. Drugs such as ranibizumab [5], aflibercept 2 mg [2], brolucizumab [6], and faricimab [7] are currently available on the market. Among these, aflibercept 2 mg is the most widely used drug. Aflibercept is a recombinant fusion protein consisting of portions of the VEGF receptor A and B extracellular domains fused to the Fc portion of human immunoglobulin G, which blocks placental growth factor (PLGF) [8].

Aflibercept 8 mg was approved as a new anti-VEGF drug in 2023 following the PULSAR phase 3 clinical trial [9]. The PULSAR trial demonstrated that injections of aflibercept 8 mg every 12 or 16 weeks were non-inferior to injections of aflibercept 2 mg every 8 weeks in terms of improvements in best-corrected visual acuity (BCVA) and central foveal thickness (CFT) [9]. Furthermore, up to week 16 of the loading phase, the fluid control effect of aflibercept 8 mg was found to be significantly superior to that of aflibercept 2 mg. Many reports have shown that switching agents can improve exudative changes in patients previously treated with anti-VEGF agents [10,11,12,13,14]. It is of particular interest to determine how effectively the newly approved aflibercept 8 mg treatment performs in this context. However, given the increasing number of drug options, it has become difficult in practice to evaluate which agent is more effective when switching treatments. If the therapeutic effect is inadequate, switching back to a previously used agent remains an option.

In this study, we retrospectively evaluated the initial response to exudative changes and the functional outcomes of intravitreal aflibercept 8 mg for nAMD in real-world settings.

## 2. Materials and Methods

This study was approved by the Ethics Committee of the Graduate School of Medicine, University of Osaka (approval number 10039), and adhered to the principles of the Declaration of Helsinki. Informed consent was not required due to the retrospective design of the study.

The study included a consecutive series of patients with nAMD who had been previously treated with other anti-VEGF drugs and switched to an intravitreal injection of aflibercept 8 mg at Osaka University Hospital between May and October 2024. Among them, patients with exudative changes (subretinal fluid [SRF], intraretinal fluid [IRF], or serous pigment epithelial detachment [sPED]) within a 6 mm × 6 mm macular cube at the time of switching to aflibercept 8 mg and who experienced no change in dosing intervals before and after the switch (within ±7 days) were included in the analysis.

All patients underwent a comprehensive eye examination, including measurement of BCVA using Landolt C charts, color fundus photography, and spectral-domain and swept-source optical coherence tomography (SD-OCT; Cirrus HD-OCT, Carl Zeiss Meditec Inc., Dublin, CA, USA and SS-OCT; DRI-SS-OCT, Topcon Inc., Tokyo, Japan) before and after switching to aflibercept 8 mg.

The outcomes included changes in BCVA, CFT (measured from the internal limiting membrane to the presumed Bruch’s membrane at the fovea) using SD-OCT and SS-OCT and exudative changes (SRF, IRF, and sPED within the macular cube) at the time of switching and the first follow-up visit. Improvement, partial improvement, or no change was assessed by three retinal specialists (CH, KS, and YF) based on the OCT findings.

### Statistical Analysis

For statistical analyses, BCVA was converted to the logarithm of the minimum angle of resolution (logMAR). One-way analysis of variance was used to assess changes in BCVA and CRT. All statistical analyses were performed using JMP Pro version 17 software (SAS Institute Inc., Cary, NC, USA). Statistical significance was set at *p* < 0.05.

## 3. Results

A total of 201 eyes from 196 patients with nAMD were switched to aflibercept 8 mg from other anti-VEGF drugs during the study period. One hundred and two eyes from 98 patients (61 male and 37 female) met the inclusion criteria (patients with exudative changes [SRF, IRF, or sPED] within a 6 mm × 6 mm macular cube at the time of switching and no difference in dosing intervals before and after the change [within ±7 days]).

The mean age was 79.6 ± 8.0 years. Forty-five eyes had polypoidal choroidal vasculopathy, forty-eight eyes had type 1 macular neovascularization (MNV), three eyes had type 2 MNV, and six eyes had type 3 MNV. The mean time from the first treatment to switching to aflibercept 8 mg was 2172 ± 1467 days, and the mean number of anti-VEGF treatments during this period was 30.8 ± 22.1 (aflibercept 2 mg: 27.8 ± 19.5; ranibizumab: 1.4 ± 4.5; faricimab: 0.9 ± 2.5; brolucizumab: 0.6 ± 2.9). Eighteen patients had a history of photodynamic therapy. Before switching to aflibercept 8 mg, 91, 5, and 6 eyes had received injections of aflibercept (2 mg), faricimab, and brolucizumab, respectively. The mean interval from the intravitreal injection immediately prior to switching to the date of switching was 62.6 ± 19.8 days (range: 28–119), and the mean interval from the date of switching to the first visit after switching was 63.7 ± 20.0 days (range: 28–126).

The logMAR BCVA at the time of the first aflibercept 8 mg injection was 0.27 ± 0.35 and remained unchanged at the first visit after switching. The CFT significantly decreased from 289 ± 115 µm to 265 ± 114 µm (*p* < 0.0001).

### 3.1. Anatomical Outcomes

**Figure 1:**
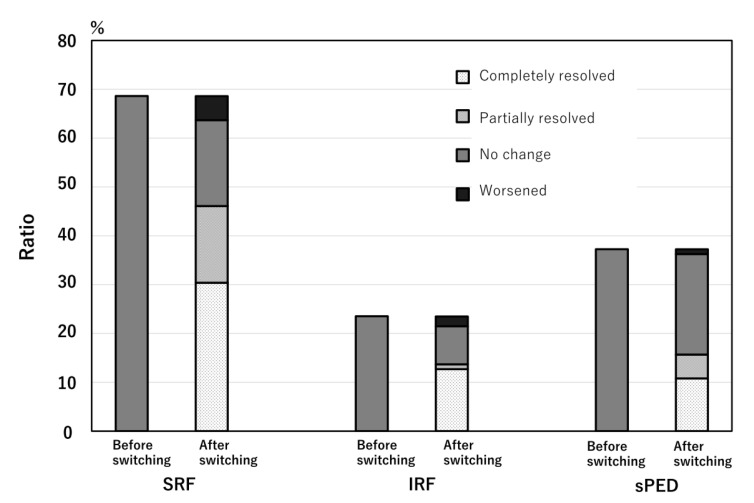
The change in the ratio of eyes with each exudative change. SRF, subretinal fluid; IRF, intraretinal fluid; sPED, serous pigment epithelium detachment.

**Figure 2:**
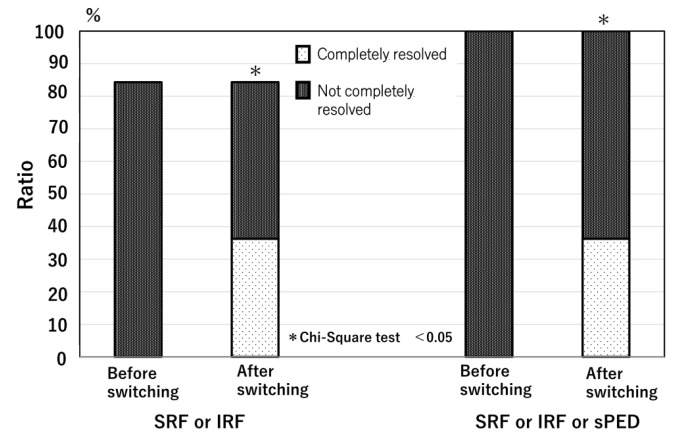
The change in the ratio of eyes with exudative change. SRF, subretinal fluid; IRF, intraretinal fluid; sPED, serous pigment epithelium detachment.

**Figure 3:**
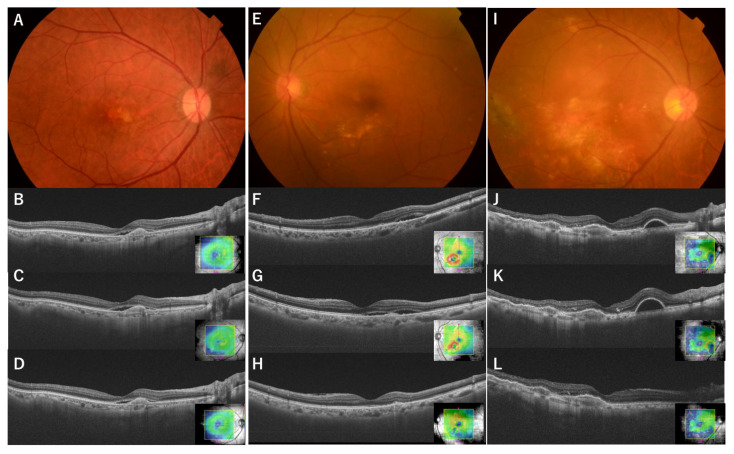
A 75-year-old man with type 2 MNV, who has been treated for 7 years and has had 28 previous intravitreal aflibercept 2 mg injections. Fundus photograph (**A**) shows a color change in the macula, and OCT on the day of the aflibercept 2 mg dose before the 8 mg switch (**B**) shows an elevation in the retinal pigment epithelium (RPE) and subretinal fluid (SRF). OCT at 63 days after the aflibercept 2 mg dose shows an increase in SRF (**C**), and aflibercept 8 mg is administered. OCT on day 63 after the 8 mg switch (**D**) shows that the SRF remains but is improved. A 72-year-old woman with polypoidal choroidal vasculopathy (PCV), who has been treated for approximately 6 years and has previously received 11 intravitreal aflibercept injections (2 mg). Fundus photography (**E**) shows a color change inferior to the macula, and OCT on the day of the aflibercept 2 mg dose before switching to 8 mg (**F**) shows SRF in the nasal inferior area. OCT 56 days after the aflibercept 2 mg dose shows SRF (**G**), and aflibercept 8 mg is administered. OCT on day 56 after the 8 mg switch shows that the SRF completely resolves (**H**). A 74-year-old man with PCV, who has been treated for 9 years and has received 33 previous intravitreal aflibercept injections (2 mg) and 3 intravitreal brolucizumab injections. Fundus photography (**I**) shows a color change in the macula and RPE atrophy. OCT on the day of brolucizumab before switching to 8 mg (**J**) shows an elevation in the RPE and serous RPE detachment (sPED) in the nasal area of the macula. OCT 70 days after the brolucizumab dose (**K**) shows an increase in sPED, and aflibercept 8 mg is administered. OCT on day 70 after the 8 mg switch (**L**) shows that the sPED completely resolves.

At the time of the initial aflibercept 8 mg injection, SRF was observed in 70 of the 102 eyes (68.6%), IRF in 24 eyes (23.5%), and sPED in 38 eyes (37.3%), within a 6 mm × 6 mm macular cube. All eyes exhibited exudative changes during switching. At the first visit after switching, SRF completely resolved in 31 of 70 eyes (44%), partially resolved in 17 eyes (24%), remained unchanged in 18 eyes (25%), and worsened in 5 eyes (7%). IRF completely resolved in 13 of 24 eyes (54%), partially resolved in 1 eye (4%), remained unchanged in 8 eyes (33%), and worsened in 2 eyes (8%). sPED completely resolved in 11 of 38 eyes (29%), partially resolved in 5 eyes (13%), remained unchanged in 21 eyes (55%), and worsened in 1 eye (3%) (Figure 1). In 53 eyes (52%), all SRF- and IRF-only exudative changes completely resolved, and in 37 eyes (36%), all exudative changes, including sPED, resolved (Figure 2 and Figure 3). There was a significant reduction in all types of exudative changes (SRF, IRF, and sPED) with *p*-values < 0.001 (chi-square test). The agreement rate between the three retinal specialists’ ratings was 98.4%.

In comparing the characteristics of cases with and without complete resolution of exudative changes, patients with complete resolution were significantly older than those without complete resolution, both in cases with either SRF or IRF or both (82.3 ± 7.3 vs. 78.1 ± 8.2; *p* = 0.012) and in cases with all exudative changes including PED (81.7 ± 7.3 vs. 78.3 ± 8.2; *p* = 0.033). Other factors (sex, year, treatment period, number of anti-VEGF treatments, MNV type, logMAR BCVA at switching, interval between injections, drug type before switching, and history of PDT) were not significantly different between patients with and without complete resolution of the exudative changes (Table 1 and Table 2).

### 3.2. Adverse Effect

No ocular or systemic adverse effects, including intraocular inflammation, were observed during the switching period to aflibercept 8 mg.

## 4. Discussion

In this study, we evaluated the initial effects of switching to aflibercept 8 mg in patients with nAMD previously treated with other anti-VEGF drugs. Several reports have investigated the therapeutic effect of switching anti-VEGF drugs [11,12,13,14,15]; however, it is difficult to accurately evaluate these effects for a number of reasons. For example, there are differences in terms of the observation and injection intervals of drug switching and whether and how often the same drug is used (especially if the effect of the switching drug is insufficient). Therefore, in the present study, to minimize bias as much as possible, the same pre- and post-dose intervals as for the previous drug were used, and exudative changes after a single dose were examined.

After switching, the mean CRT significantly decreased, and exudative changes significantly improved. SRF and IRF completely resolved in approximately half of the eyes, and PED resolved in 30% of eyes. In the PULSAR study [9], compared to patients treated with aflibercept 2 mg, the percentage of patients treated with aflibercept 8 mg who had no exudative changes at 16 weeks (8 weeks after the loading dose) was 51.6% for 2 mg and 63.3% for 8 mg, significantly higher than for 2 mg. The effectiveness of high-molar anti-VEGF agents has previously been reported. In the MARINA and ANCHOR trials, ranibizumab 0.5 mg showed an advantage over 0.3 mg in terms of its functional and anatomical effects [5,16]. The HAWK trial also showed similar results, with brolucizumab 6 mg reporting better anatomical outcomes compared to 3 mg brolucizumab [6,17].

In the treatment of nAMD with a treat-and-extend regimen, the treatment interval cannot be extended for patients with residual exudative changes despite continuous and frequent administration of anti-VEGF drugs [18]. Although simple comparisons cannot be made, it is expected that fewer cases of exudation will remain even after a short time. The patients in this study were predominantly those with an average dosing interval of 62.6 days (approximately 9 weeks), making it difficult to extend the dosing interval. The disappearance of exudative changes in approximately half of the cases suggests that a switch to aflibercept 8 mg may extend the dosing interval. In the ALTIR study, which examined the aflibercept 2 mg dosing interval [19], approximately 40% of cases were treated with the shortest interval of 8 weeks; in contrast, in the PULSAR trial, which was a phase 3 clinical trial of aflibercept 8 mg, only about 15% of cases were treated with 8-week intervals [9]. Although a simple comparison between these two trials cannot be made, the use of aflibercept 8 mg is expected to reduce the number of cases with residual exudative changes after repeated short-term treatment.

In this study, all types of exudative changes significantly disappeared after treatment with aflibercept 8 mg, and SRF and IRF improved in approximately half of the cases, whereas sPED improved in approximately 30% of cases. A previous report of a large number of patients who switched from 2 mg to 8 mg reported significant improvement in SRF and IRF after three doses, but no significant difference in PED [15]. This result is similar to the trend observed in this study, and improvement can be expected for IRF and SRF.

Additionally, this study revealed that the patients with complete resolution of exudative changes were significantly older than those without. The difference is small (81.7 ± 7.3 vs. 78.3 ± 8.2), and the reason is unclear. However, older patients may have fewer exudative changes dependent on the hyperpermeability of choroidal vessels, such as pachychoroid diseases, and more VEGF-dependent exudative changes [20,21,22].

In this study, no systemic or ocular adverse effects were observed. It should be noted that the observation period in this study was only during follow-up after the first treatment. Several reports call for caution regarding intraocular inflammation after switching to aflibercept 8 mg [11,15,23,24,25]. Considering that the intraocular inflammation in these reports was also mild in all cases and that no intraocular inflammation was observed in this study, the incidence of intraocular inflammation is low, and the risk of vision loss due to intraocular inflammation induced by switching to aflibercept 8 mg is considered to be very low due to the mild nature of the disease. However, some reported cases developed intraocular inflammation after the second or third injection of aflibercept 8 mg [23,24], and further attention should be paid to these cases.

The main limitations of this study are its retrospective, single-center nature and short-term outcomes. In addition, only the response to the first treatment was evaluated, and the prolongation of treatment intervals and the durability of treatment were not evaluated. Furthermore, the non-comparative nature of this study introduces a selection bias and does not control for regression toward the mean; all participants were Japanese. However, to minimize any bias in this study, such as treatment intervals or discontinuation of therapy due to inadequate efficacy, only cases with equal intervals before and after the first injection upon switching to aflibercept 8 mg were included. The anatomical response after the first injection of a new drug has a significant effect on whether treatment can be continued thereafter. In this cohort of patients, switching to aflibercept 8 mg significantly reduced the presence of SRF, IRF, and PED and improved CRT after the first injection. Although further prospective studies with larger sample sizes should involve long-term outcomes, this study is meaningful in evaluating the effects of switching from 2 to 8 mg in the real world over a short period.

## 5. Conclusions

In conclusion, switching from other anti-VEGF drugs to aflibercept 8 mg in patients with nAMD significantly reduced exudative changes, even after a single dose, and was particularly effective against SRF and IRF.

## Figures and Tables

**Table 1:** The characteristics of the cases with or without complete resolution of exudative changes (SRF, IRF, or both).

	Complete Resolution (n = 38)	Not Complete Resolution (n = 43)	*p*-Value
Age (mean ± SD)	82.3 ± 7.3	78.1 ± 8.2	0.012 *
Gender (male %)	24 (63%)	29 (59%)	0.706
MNV type (PCV/type 1/type 2/type 3)	15/18/1/4	20/25/2/2	0.685
Treatment period (days)	2217 ± 1400	2078 ± 1537	0.664
Number of previous anti-VEGF treatments	31.6 ± 22.4	29.0 ± 22.5	0.599
Number of previous aflibercept 2 mg treatments	29.6 ± 20.6	26.0 ± 19.4	0.405
History of PDT (yes: n (%))	5 (13%)	9 (18%)	0.512
Treatment interval at switching	64.7 ± 22.8	62.2 ± 14.4	0.542
Anti-VEGF drug at switching (aflibercept/faricimab/brolucizumab)	37/0/1	43/4/2	0.178

SRF, subretinal fluid; IRF, intraretinal fluid; PCV, polypoidal choroidal vasculopathy; VEGF, vascular endothelial growth factor; PDT, photodynamic therapy. * *p*-value < 0.05

**Table 2:** The characteristics of the cases with or without complete resolution of exudative changes (all types of exudative changes).

	Complete Resolution (n = 37)	Not Complete Resolution (n = 65)	*p*-Value
Age (mean ± SD)	81.7 ± 6.8	78.3 ± 8.2	0.033
Gender (male %)	26 (70%)	37 (57%)	0.180
MNV type (PCV/type 1/type 2/type 3)	14/19/1/3	31/29/2/3	0.742
Treatment period (days)	2412 ± 1317	2036 ± 1540	0.215
Number of previous anti-VEGF treatments	35.2 ± 22.4	28.2 ± 21.7	0.123
Number of previous aflibercept 2 mg treatments	32.8 ± 20.7	25.0 ± 18.4	0.054
History of PDT (yes: n (%))	6 (16%)	7 (17%)	0.926
Treatment interval at switching	63.4 ± 21.6	62.1 ± 18.8	0.757
Anti-VEGF drug at switching (aflibercept/faricimab/brolucizumab)	34/1/2	57/4/4	0.725

SRF, subretinal fluid; IRF, intraretinal fluid; PCV, polypoidal choroidal vasculopathy; VEGF, vascular endothelial growth factor; PDT, photodynamic therapy.

## Data Availability

All data generated or analyzed during this study are included in this published article.

## Abbreviations

The following abbreviations are used in this manuscript:

nAMD	neovascular age-related macular degeneration
VEGF	vascular endothelial growth factor
PLGF	placental growth factor
BCVA	best-corrected visual acuity
CFT	central foveal thickness
SRF	subretinal fluid
IRF	intraretinal fluid
sPED	serous pigment epithelial detachment
OCT	optical coherence tomography
logMAR	logarithm of the minimum angle of resolution
MNV	macular neovascularization
